# Effect of Chloride Concentration on the Corrosion Behavior of an Iron-Based Amorphous Coating and 316L Stainless Steel in Saline Soil from Daqing

**DOI:** 10.3390/ma19143093

**Published:** 2026-07-18

**Authors:** Na Xu, Guangci Li, Yong Wang

**Affiliations:** 1 College of Chemical and Mechanical Engineering, Liaodong University, 116 Linjiang Back Street, Dandong 118001, China; manmanhappytu@126.com; 2 School of Mechanical Science and Engineering, Northeast Petroleum University, 199 Fazhan Road, Daqing 163318, China

**Keywords:** iron-based amorphous coating, 316L stainless steel, soil corrosion, chloride ion, pitting, electrochemical impedance spectroscopy

## Abstract

AISI 316L stainless steel (316L SS) exhibits inadequate corrosion resistance in chloride-containing soils. Fe-based amorphous coatings (Fe-ACs), owing to their high Cr, Mo, and W contents and defect-free amorphous structure, are promising candidates for superior protection. In this work, the corrosion behavior of 316L SS and an Fe-based amorphous coating (Fe-AC) fabricated by high-velocity oxygen-fuel (HVOF) spraying was systematically compared by burial in Daqing saline soil (25% water content) with 0, 1.0, and 2.0 wt.% NaCl for 15–55 days. Corrosion rates were measured via mass loss, and surface morphology, elemental distribution, and phase constitution were characterized using OM, SEM/EDS, and XRD. Electrochemical impedance spectroscopy and potentiodynamic polarization were employed to assess passive-film stability and charge-transfer resistance. The Fe-AC consistently exhibited an extremely low corrosion rate (below 0.01 mm y^−1^), nearly independent of NaCl concentration and exposure time, with only sporadic rust spots and the formation of a compact Cr/Mo/W-enriched passive film. In contrast, after 55 days in soil containing 2.0 wt.% NaCl, the 316L SS showed a corrosion rate of 0.0562 mm y^−1^—six times that of the Fe-AC—accompanied by severe pitting (pit depth up to 3.6 mm) and loose corrosion products (γ-FeOOH and α-Fe_2_O_3_). Electrochemical tests confirmed that the charge-transfer resistance of the Fe-AC under the 0% NaCl condition reached 1.16 × 10^6^ Ω cm^2^ and its breakdown potential exceeded 1.12 V, far outperforming 316L SS (2.30 × 10^3^ Ω cm^2^ and 0.22 V, respectively). The novelty of this study lies in the systematic evaluation of the buried corrosion performance of HVOF-sprayed Fe-based amorphous coatings versus 316L SS in an actual saline soil and in elucidating the synergistic passivation mechanism of Cr, Mo, and W. This passive film effectively impedes chloride ingress and maintains high impedance over extended periods.

## 1. Introduction

Metal corrosion causes severe economic losses—representing 2.5–4.2% of GDP in major countries and over 4% in China—while also threatening operational safety via equipment leakage [1]. Among these, buried metallic structures—such as oil and gas transmission pipelines, oil-water well casings, and grounding grids—are particularly vulnerable; owing to their long-term service in complex soil environments, their corrosion-induced failure represents a persistent and critical challenge in the petroleum and infrastructure sectors [2]. As a porous medium comprising solid, liquid, and gas phases, soil exhibits corrosivity that is synergistically influenced by multiple factors, including moisture content, pH, salinity, aeration, and microbial activity [3,4]. The physicochemical properties of soils vary substantially across regions. The Daqing Oilfield, a major petroleum production base in China, features largely saline–alkali soils, with pH values ranging from 7.17 to 10.3 and elevated concentrations of corrosive anions such as Cl^−^, SO_4_^2−^, and HCO_3_^−^ [5]. Corrosivity assessments indicate that most soils in this area fall into the highly corrosive category [6], and corrosion of underground equipment has exhibited a clear accelerating trend. For example, at one Daqing production plant, among the more than 120 leaking wells whose casings have been retrieved in recent years, wells equipped with electric submersible pumps (ESPs) accounted for approximately 50–60% [7]. Therefore, developing surface protection technologies for pipeline materials suited to highly corrosive soil environments holds considerable engineering significance for extending the service life of underground facilities and safeguarding production safety in oilfields.

Substantial research efforts have been directed toward understanding the corrosion of metallic materials in soil environments. The corrosion rate is governed by factors including moisture content, pH, soluble salt content, porosity, and microbial activity, among which dissolved oxygen and chloride ions are recognized as the most critical promoters [3,4,8]. Owing to their small ionic radius and high penetrating power, chloride ions preferentially adsorb at defect sites on the passive film. Through competitive adsorption with oxygen atoms, they induce localized dissolution and create an autocatalytic acidification environment, thereby facilitating pit nucleation and growth [9,10]. Studies have shown that chloride media significantly increase the pitting susceptibility of stainless steels, as manifested by a decrease in pitting potential and a deterioration in passive film stability [11,12]. Moreover, multiple anions (Cl^−^, SO_4_^2−^, HCO_3_^−^) act synergistically in soil corrosion: Cl^−^ primarily disrupts the passive film and triggers pitting, whereas HCO_3_^−^ promotes anodic dissolution at low concentrations but may form a protective layer at high concentrations [13,14]. Qin et al. [15] confirmed through direct evidence from soil comparisons across different regions of Myanmar that the corrosion rate of X70 pipeline steel increases sharply with rising soil chloride content (0.008–0.246%), attributable to chloride penetration of the passive film and enhanced solution conductivity. Furthermore, Akkouche et al. [16] revealed that in chloride-containing unsaturated soils, even though the macroscopic average corrosion rate of S235JR carbon steel decreases with reduced moisture content, chloride-induced local active zones persist and can generate localized perforation rates up to 1.5 mm/a under oxygen concentration-cell effects.

Fe-based amorphous alloy coatings are surface-protective materials characterized by a unique non-crystalline structure. Owing to their high strength, high hardness, excellent wear and corrosion resistance, and relatively low cost, they have garnered widespread attention in surface engineering in recent years [17,18]. Studies indicate that Fe-Cr-Mo-based amorphous coatings can form highly stable passive films in chloride-containing solutions, featuring a wide passivation range and high breakdown potential, and their pitting resistance surpasses that of conventional stainless steels [19,20]. Wang et al. [20] reported that the transpassive potential of high-velocity oxygen-fuel (HVOF)-sprayed Fe-based amorphous coatings (AMCs) in sand-containing NaCl solution is approximately 1.2 V, significantly higher than that of 304 stainless steel (0.4 V), thus demonstrating superior localized corrosion resistance. Additionally, passive films enriched with Mo and W can effectively enhance resistance to Cl^−^ attack. The superior corrosion resistance of Fe-based amorphous coatings stems from two aspects. First, the amorphous structure provides an absence of grain boundaries, dislocations, and other corrosion-sensitive microstructural features, making it difficult for aggressive ions such as Cl^−^ to penetrate through structural defects, thereby substantially inhibiting the initiation and propagation of corrosion [21]. Second, the high content of alloying elements such as Cr, Mo, and W undergoes selective oxidation during corrosion, forming a dense and stable oxide film rich in these elements that effectively blocks Cl^−^ penetration [21,22]. However, existing studies have mostly been conducted in idealized electrolyte environments such as 3.5 wt.% NaCl solution and H_2_SO_4_ solution [17,18,23], and the experimental conditions (immersion or salt-spray tests) differ considerably from actual soil-buried service conditions. Consequently, systematic experimental studies on the long-term buried corrosion behavior of Fe-based amorphous coatings in saline–alkali soils, their corrosion performance compared with the 316L substrate, and their failure mechanisms in authentic soil environments remain lacking [24].

This study systematically evaluates the buried-soil corrosion performance of high-velocity oxygen-fuel (HVOF)-sprayed Fe-based amorphous coatings and 316L stainless steel in actual saline–alkali soils, and elucidates the synergistic passivation mechanism involving Cr, Mo, and W, by which the passive film effectively impedes chloride ingress and maintains high impedance over extended periods. Following preparation of the Fe-based amorphous coatings via HVOF, a comparative investigation was conducted on the corrosion behavior of the coatings versus 316L stainless steel buried in soils containing varying NaCl concentrations (0, 1.0, and 2.0 wt.%) for durations of 15 to 55 days. Corrosion resistance was evaluated through weight-loss measurements, surface morphology and phase analysis (SEM/EDS and XRD), and electrochemical tests (EIS and potentiodynamic polarization), while the underlying corrosion mechanisms were also elucidated. This work aims to provide a novel materials-based foundation for the protection of metallic components in saline–alkali soil environments.

## 2. Experimental Materials and Methods

### 2.1. Experimental Materials

**Figure 1:**
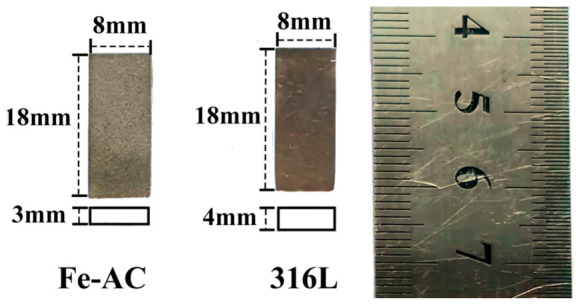
Dimensions of corrosion test coupons.

The experimental materials consisted of bare 316L stainless steel (316L SS) (purchased from Shanghai Qianxiang Stainless Steel Co., Ltd., Shanghai, China) and 316L SS substrates coated with an iron-based amorphous coating prepared by high-velocity oxygen-fuel (HVOF) spraying (hereafter referred to as Fe-AC), for which the coating powder was supplied by Ningbo Zhongke Hongjing New Material Technology Co., Ltd. (Ningbo, China). The chemical composition of the coating (mass fraction, wt.%) was Fe_54.2_, Cr_18.3_, Mo_13.7_, Mn_2.0_, W_6.0_, B_3.3_, C_1.1_, and Si_1.4_. The geometric dimensions of the corrosion test coupons are shown in Figure 1. It should be noted that the cutting and machining processes used in specimen preparation may introduce localized cold working and strain hardening on the edges and surfaces of the specimens, both of which can serve as preferential initiation sites for pitting corrosion. The test soil was collected from the Daqing Oilfield region in China (its physicochemical properties are listed in Table 1). The soil media were prepared by air-drying, grinding, and sieving, followed by the addition of deionized water and NaCl to achieve a moisture content of 25 wt.% and NaCl concentrations of 0, 1.0, and 2.0 wt.% relative to the dry soil mass, respectively. Bare 316L SS and Fe-AC specimens were buried in the prepared soils and retrieved after 15, 25, 35, 45, and 55 days. Subsequently, the corrosion products were removed via chemical cleaning using a solution consisting of 10 g hexamethylenetetramine, 100 mL concentrated hydrochloric acid, and 1 L deionized water. The cleaning procedure was conducted at ambient temperature, with immersion times of 5 min for Fe-AC and 10 min for 316L SS, after which the specimens were sequentially rinsed with deionized water and ethanol and then dried. At each time interval and for each NaCl concentration, five parallel specimens were employed for weight-loss measurements and corrosion rate calculations. The corrosion rate was determined using Equation (1):

(1) $$\;v\;=\;\frac{\;\Delta m}{A\cdot \rho \cdot t}$$

where *v* is the average corrosion rate (mm/a), Δ*m* is the mass loss of the specimen before and after corrosion (g), *A* is the surface area of the specimen exposed to the soil (cm^2^), ρ is the density of the specimen material (g/cm^3^), and t is the burial time (h). The result was then converted to millimeters per year (mm/a).

The corrosion morphologies on the specimen surfaces were examined using an optical microscope (OM, GX71, Evident, Tokyo, Japan). The microstructural features and elemental distributions of the corrosion products were characterized by field-emission scanning electron microscopy (SEM, JSM-7800F, JEOL, Tokyo, Japan) equipped with an energy-dispersive X-ray spectrometer (EDS). The phase compositions of the corrosion products were analyzed via X-ray diffraction (XRD, X’Pert Pro, Panalytical, Almelo, The Netherlands). The depth and width of corrosion pits were measured using ImageJ 1.54m image analysis software. In addition, the surface morphologies of the electrodes after potentiodynamic polarization testing were also observed (the electrochemical measurements were performed with a PARSTAT 2273 electrochemical workstation, Princeton Applied Research, Oak Ridge, TN, USA).

### 2.2. Electrochemical Measurements

**Figure 2:**
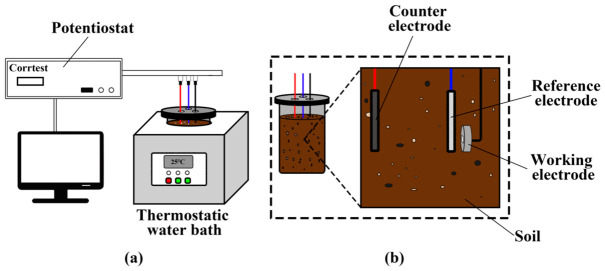
Electrochemical corrosion testing setup: (**a**) electrochemical workstation; (**b**) three-electrode test system in the soil medium.

Electrochemical measurements were performed using a PARSTAT 2273 electrochemical workstation with a standard three-electrode system. The working electrode was the test specimen (exposed area of 1.0 cm^2^), the counter electrode was a platinum sheet, and the reference electrode was a saturated calomel electrode (SCE). The test temperature was maintained at 298 K. The electrolyte was the soil medium with a water content of 25% prepared with NaCl solutions at concentrations of 0, 0.5, 1.0, 1.5, and 2.0 wt.% (relative to the dry soil mass). Before testing, the specimens were sequentially ground with 1200# SiC abrasive paper to achieve a uniform surface, then ultrasonically degreased in acetone, rinsed with deionized water, and air-dried at room temperature. The treated specimens were immersed in the electrolyte and allowed to stabilize for 1 h to ensure a stable open-circuit potential (OCP). Potentiodynamic polarization scans were then conducted over a potential range from −500 mV to +1500 mV relative to OCP at a scan rate of 0.33 mV/s. Electrochemical impedance spectroscopy (EIS) was performed at OCP over a frequency range of 100 kHz to 10 mHz with an AC excitation signal amplitude of 10 mV. The obtained EIS data were fitted to an equivalent circuit using ZView 3.0 software.. All electrochemical tests were repeated at least three times to ensure reproducibility and statistical reliability. The electrochemical testing process is illustrated in Figure 2.

## 3. Results and Discussion

### 3.1. Corrosion Rate and Weight Loss Analysis

**Figure 3:**
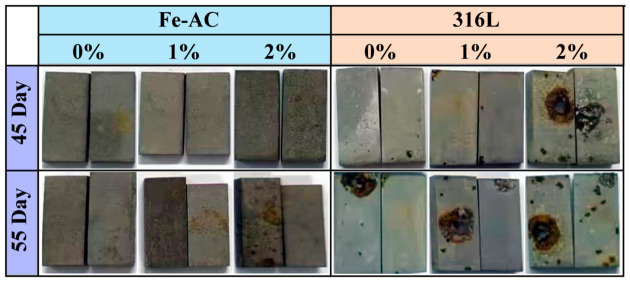
Morphologies of corrosion coupons after 45 and 55 days of burial.

Figure 3 presents the macroscopic corrosion morphologies of Fe-AC and 316L SS specimens retrieved from soils with varying NaCl concentrations at the 45- and 55-day sampling intervals. No visible corrosion was observed on any specimens at the 15-, 25-, and 35-day samplings; therefore, only the 45- and 55-day results are shown.

For Fe-AC buried in soil containing 2.0 wt.% NaCl, sparsely distributed dark-brown rust spots appeared on the surface after 45 days. These spots remained relatively small and isolated, without coalescing into continuous patches. By day 55, the number of rust spots had increased, although their size did not appreciably enlarge; most of the surface still retained a metallic luster, and no distinct pitting or exposed substrate was observed. In the 0 and 1.0 wt.% NaCl soils, only a very limited number of rust spots were visually detectable on the Fe-AC surfaces.

For 316L SS buried in the 2.0 wt.% NaCl soil for 45 days, the surface exhibited extensive, continuous reddish-brown corrosion product layers. The central region showed notably dark and concave features, indicative of severe localized attack. In the 0 and 1.0 wt.% NaCl soils at 45 days, surface rust was relatively sparse but still locally visible. By day 55, 316L SS developed thick, extensive reddish-brown corrosion product deposits under all three soil conditions (0, 1.0, and 2.0 wt.% NaCl). The dark, pit-like features in the central region became more pronounced and darker in color, suggesting that localized corrosion had progressed in the depth direction over time. Comparing the two materials, the corrosion morphology of Fe-AC was characterized by discrete, self-limiting surface rust spots without any pitting. In contrast, 316L SS exhibited continuous, loose corrosion product layers superimposed on central dark concavities, indicative of a distinct localized-pitting evolutionary process.

**Figure 4:**
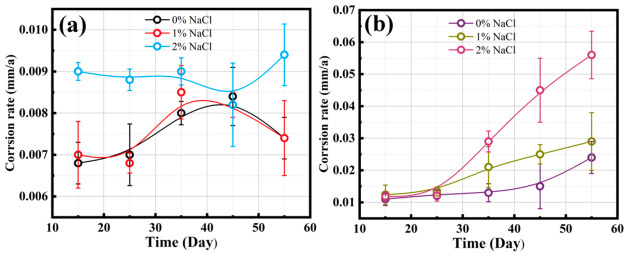
Average corrosion rate as a function of burial time (15–55 days) for specimens buried in soils with different NaCl additions (0, 1.0, 2.0 wt.%): (**a**) Fe-AC; (**b**) 316L SS.

It is important to point out that the visual onset time of macroscopic rust spots in Figure 3 does not coincide with the passive film formation time. The steady low rate observed in Figure 4a indicates that the passive film had already developed within 15 days; hence, the belated emergence of rust spots arises from inadequate build-up of early corrosion products and sluggish soil mass transfer, not from a retarded passivation process.

Figure 4 presents the average corrosion rates of Fe-AC and 316L SS as a function of burial time (15–55 days) in soils containing 0, 1.0, and 2.0 wt.% NaCl; the numerical values are listed in Table 2. For the Fe-AC (Figure 4a), the corrosion rate remained consistently low (0.0068–0.0094 mm/a) throughout the entire burial period, showing no clear increasing trend with time, regardless of the NaCl concentration. In the 0 wt.% NaCl soil, the corrosion rate fluctuated slowly from 0.0068 mm/a at 15 days to 0.0074 mm/a at 55 days, suggesting the formation of a stable protective oxide film on the coating surface. As the NaCl concentration increased to 1.0 wt.% and 2.0 wt.%, the corrosion rate rose slightly, but the increase was limited (the highest value was 0.0094 mm/a at 2.0 wt.% NaCl). These results indicate that the Fe-AC has good tolerance to chloride ions and does not exhibit obvious pitting-induced accelerated corrosion.

In contrast, 316L SS (Figure 4b) showed significantly higher corrosion rates than the Fe-AC, and the rates increased markedly with both burial time and NaCl concentration. In the 0 wt.% NaCl soil, the corrosion rate gradually increased from 0.0112 mm/a at 15 days to 0.0246 mm/a at 55 days, indicating that even in chloride-free soil, 316L SS undergoes sustained uniform corrosion. In the 1.0 wt.% and 2.0 wt.% NaCl soils, the corrosion rate exhibited an accelerating trend. Notably, under the 2.0 wt.% NaCl condition, the rate rose sharply from 0.0117 mm/a at 15 days to 0.0562 mm/a at 55 days—approximately six times that of the Fe-AC under the same conditions. This behavior is attributed to the breakdown of the passive film on the stainless steel surface by chloride ions: Cl^−^ preferentially adsorbs at defects in the passive film, induces pitting nucleation, and as the burial time extends, the pits expand in both depth and lateral direction, leading to a marked increase in the overall corrosion rate [25,26].

Notably, during the early stage of burial (15–35 days), the corrosion rates of 316L SS in the 1.0 wt.% and 2.0 wt.% NaCl soils were relatively close (e.g., 0.0134 and 0.0121 mm/a, respectively, at 25 days). After 35 days, however, the difference between the two conditions rapidly widened, and by 55 days, the corrosion rate under 2.0 wt.% NaCl (0.0562 mm/a) was approximately 1.9 times that under 1.0 wt.% NaCl (0.0296 mm/a). This result demonstrates that the accelerating effect of chloride ions on 316L SS corrosion is strongly concentration-dependent and exhibits a time-accumulation effect.

The Fe-AC coating shows far superior corrosion resistance to 316L SS in chloride-containing soil environments. Its corrosion rate consistently remains below 0.01 mm/a and is only weakly affected by chloride concentration. In contrast, the corrosion rate of 316L SS increases significantly in the presence of chloride ions, approaching a highly corrosive level after 55 days of burial in the 2.0 wt.% NaCl soil. Overall, the corrosion rate of the Fe-AC specimen is substantially lower than that of 316L SS, indicating that the iron-based amorphous coating provides excellent inhibition of localized corrosion in soil environments.

### 3.2. Corrosion Morphology and Phase Analysis of Corrosion Products

**Figure 5:**
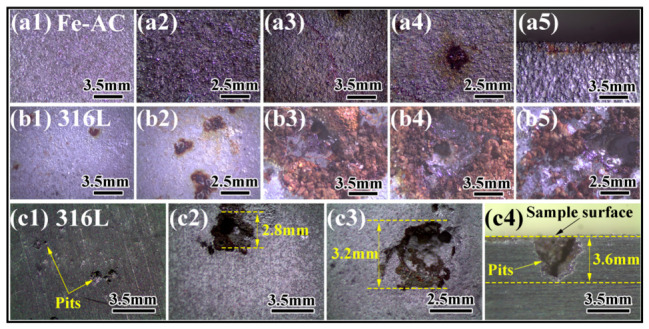
Corrosion morphologies of different specimens: (**a1**–**a5**) iron-based amorphous coating (Fe-AC); (**b1**–**b5**) 316L stainless steel (before cleaning); (**c1**–**c4**) 316L stainless steel (after cleaning).

Figure 5 shows the macroscopic morphologies of the corrosion coupons after 55 days of burial. As shown in Figure 5(a1–a5), the Fe-AC surface exhibited dark red rust spots, and linear reddish-brown rust stains were observed along one edge of the specimen. In contrast, the 316L SS surface (Figure 5(b1–b5)) displayed clear corrosion pits surrounded by accumulations of reddish-brown corrosion products. After cleaning (Figure 5(c1–c4)), the pit widths were measured to be approximately 2.8 mm and 3.2 mm, and the pit depth reached up to 3.6 mm, indicating that 316L SS underwent severe localized corrosion in the chloride-containing soil.

**Figure 6:**
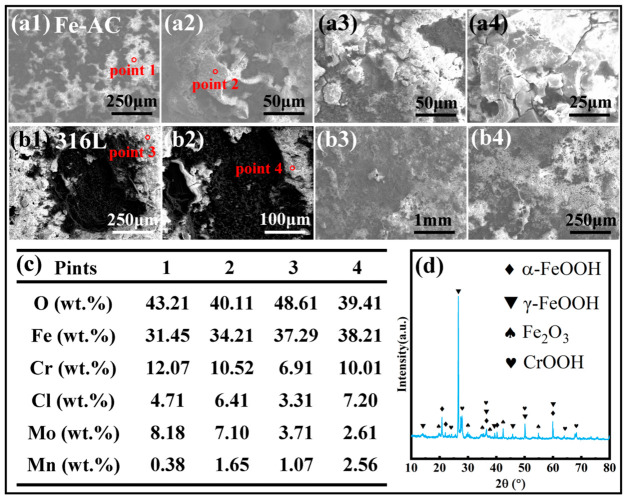
Morphology and phase characterization of corrosion products: (**a1**–**a4**) Fe-AC; (**b1**–**b4**) 316L SS; (**c**) EDS; (**d**) XRD.

Figure 6 presents the microstructure and phase composition of the corrosion products. Scanning electron microscopy (SEM) observation (Figure 6(a1–a4)) revealed that the corrosion products on the Fe-AC surface exhibited a shell-like structure covering the specimen surface and the interspaces between unmelted particles, and the structure was relatively dense. In contrast, the products around the corrosion pits on 316L SS (Figure 6(b1–b4)) were loose and porous, with poor compactness, making them ineffective at blocking the penetration of corrosive media. The EDS analysis (Figure 6c) showed that the corrosion products on the Fe-AC surface mainly contained Fe, Cr, and O, along with small amounts of alloying elements such as Mo and W. The mass fraction of Cr was markedly higher than the nominal composition of the coating, indicating that chromium enrichment occurred during corrosion, which favors the formation of a dense Cr-rich oxide film. On the 316L SS surface, the corrosion products consisted primarily of Fe and O, with a relatively low Cr content, and no obvious enrichment of alloying elements was observed.

Because the amount of corrosion products on the Fe-AC surface was very small and insufficient for XRD analysis, only the corrosion products on the 316L SS surface were characterized by XRD. As shown in Figure 6d, the XRD pattern of the 316L SS corrosion products mainly exhibited characteristic diffraction peaks of α-Fe_2_O_3_ (hematite) and γ-FeOOH (lepidocrocite), which is consistent with the corrosion products reported in previous studies [26,27]. γ-FeOOH is a loose and unstable iron oxyhydroxide; its presence further confirms that the corrosion products on the 316L SS surface are poorly protective, allowing chloride ions to penetrate and initiate pitting. This result is highly consistent with the high corrosion rate of 316L SS and its severe localized corrosion morphology (Figure 5(b1–b5,c1–c4)). For the Fe-AC, the limited amount of corrosion products suggests that the Cr-rich passive film formed on its surface is extremely thin and dense, thereby effectively suppressing further product formation—a key reason why its corrosion rate is significantly lower than that of 316L SS.

In summary, the synergistic effect of the high Cr, Mo, and W contents in the iron-based amorphous coating induces the formation of a dense and stable Cr-rich oxide film, which effectively inhibits the attack of Cl^−^ on the substrate. In contrast, the corrosion products formed on 316L SS in the chloride-containing soil environment are loose and poorly protective, enabling chloride ions to penetrate and promote the nucleation and propagation of pitting. This result is in good agreement with the corrosion rate data (Figure 4 and Table 2).

### 3.3. Electrochemical Corrosion Behavior

**Figure 7:**
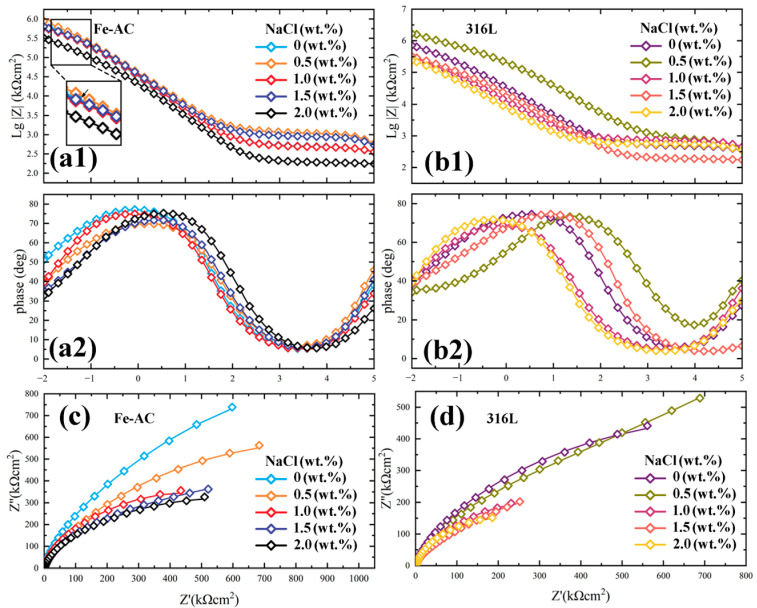
Electrochemical impedance spectra of different specimens: (**a1**,**a2**) Bode plots of Fe-AC; (**b1**,**b2**) Bode plots of 316L SS; (**c**) Nyquist plots of Fe-AC; (**d**) Nyquist plots of 316L SS.

**Figure 8:**
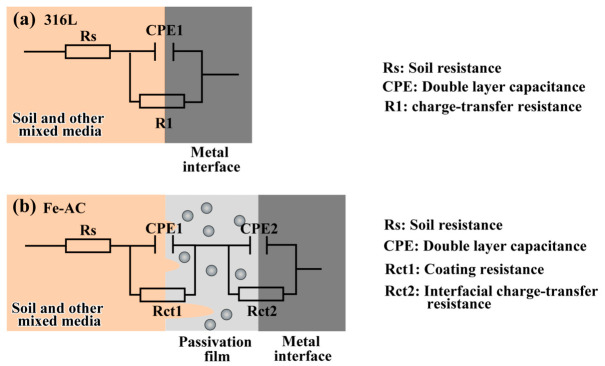
Equivalent circuits used for fitting the EIS data: (**a**) 316L SS; (**b**) Fe-AC.

Figure 7 shows the Nyquist and Bode plots of Fe-AC and 316L SS in soils with different NaCl contents. To further quantitatively evaluate the corrosion behavior, the EIS data were fitted using equivalent circuits. As illustrated in Figure 8, the EIS data of Fe-AC were fitted with a two-time-constant equivalent circuit, Rs (Q1 (R1 (Q2R2))), and the fitted parameters are listed in Table 3. The EIS data of 316L SS were fitted with a one-time-constant equivalent circuit, R_s_ (QdlRct), and the fitted parameters are listed in Table 4.

For the Fe-AC coating, the two-time-constant model was used for fitting (Table 3). The high-frequency time constant (CPE1, Rct1) reflects the dielectric and resistive characteristics of the coating itself, whereas the low-frequency time constant (CPE2, Rct2) corresponds to the charge transfer process at the coating/metal interface. As the NaCl content increased, Rs decreased from 1144 Ω·cm^2^ to 494.4 Ω·cm^2^, a trend consistent with that observed for 316L SS. The high-frequency resistance Rct1 gradually decreased from 63.06 × 10^4^ Ω·cm^2^ to 34.09 × 10^4^ Ω·cm^2^, indicating that chloride ions penetrated into the micropores of the coating, causing some degradation of its barrier performance.

From Table 4, as the NaCl content in the soil increased from 0 to 2.0 wt.%, the solution resistance Rs of 316L SS gradually decreased from 1115.2 Ω·cm^2^ to 797.4 Ω·cm^2^, which is attributed to the increased ionic conductivity of the soil electrolyte due to the higher chloride concentration. Meanwhile, the Y0 value of the constant phase element CPEdl increased significantly from 1.85 × 10^−^^5^ Ω^−^^1^·cm^−^^2^·s^−^^n^ to 9.46 × 10^−^^4^ Ω^−^^1^·cm^−^^2^·s^−^^n^, an increase of approximately 50-fold, while the exponent *n* remained between 0.80 and 0.84. This indicates a marked increase in the double-layer capacitance at the electrode surface, likely associated with increased surface roughness or the formation of a corrosion product layer. More critically, the charge transfer resistance Rct (denoted as R1 in Table 4) showed a continuous decreasing trend with increasing NaCl content, dropping from 10.80 × 10^3^ Ω·cm^2^ at 0 wt.% NaCl to 2.30 × 10^3^ Ω·cm^2^ at 2.0 wt.% NaCl. The decrease in Rct directly reflects the enhanced destructive effect of chloride ions on the passive film of 316L SS, reducing the resistance to anodic dissolution and thereby increasing the corrosion rate. This result is in good agreement with the corrosion rate data (Figure 4 and Table 2), indicating that the corrosion resistance of 316L SS deteriorates significantly in chloride-containing soil.

Additionally, for the Fe-AC coating, the Y0 of CPE1 increased from 5.46 × 10^−^^6^ to 2.74 × 10^−^^5^ Ω^−^^1^·cm^−^^2^·s^−^^n^, and n1 decreased from 0.78 to 0.64, suggesting an increase in coating capacitance and porosity. Nevertheless, the coating still maintained a relatively high resistance (≥3.4 × 10^5^ Ω·cm^2^). The low-frequency charge transfer resistance Rct2 is the key parameter for evaluating the corrosion resistance of the coating. In the soil with 0 wt.% NaCl, Rct2 was as high as 116.09 × 10^4^ Ω·cm^2^ (approximately 1.16 × 10^6^ Ω·cm^2^), indicating that the interfacial corrosion reaction was greatly suppressed and the coating provided excellent protection to the substrate. As the NaCl content increased to 1.0 wt.%, Rct2 decreased to 24.24 × 10^4^ Ω·cm^2^; at 1.5 wt.% NaCl, it rebounded slightly to 35.51 × 10^4^ Ω·cm^2^, which may be attributable to local self-repair of the passive film or test variability. However, under the 2.0 wt.% NaCl condition, Rct2 dropped sharply to 0.65 × 10^4^ Ω·cm^2^ (i.e., 6.5 × 10^3^ Ω·cm^2^), which is of the same order of magnitude as, but slightly higher than, the Rct of 316L SS under the same condition (2.30 × 10^3^ Ω·cm^2^). This indicates that in a high-chloride environment, the interfacial protective capability of the Fe-AC coating is markedly weakened, though it remains marginally better than that of bare 316L SS. Furthermore, the Y0 of CPE2 increased from 4.33 × 10^−^^6^ to 6.70 × 10^−^^4^ Ω^−^^1^·cm^−^^2^·s^−^^n^, and n2 increased from 0.62 to 0.75, reflecting an increase in double-layer capacitance and a greater number of active corrosion sites.

Comparing Table 3 and Table 4, under low-chloride conditions (0–1.0 wt.% NaCl), the Rct2 of Fe-AC is higher by one to two orders of magnitude than that of 316L SS, indicating that the iron-based amorphous coating exhibits significantly superior corrosion resistance in chloride-containing soil. Even under high-chloride conditions (2.0 wt.% NaCl), the Rct2 of Fe-AC (6.5 × 10^3^ Ω·cm^2^) remains slightly higher than the Rct of 316L SS (2.30 × 10^3^ Ω·cm^2^), and the coating resistance Rct1 of Fe-AC is still maintained above 3.4 × 10^5^ Ω·cm^2^, suggesting that the coating itself still provides a certain barrier effect. Therefore, the EIS quantitative analysis confirms that the Fe-AC coating has better corrosion resistance than 316L SS in salinized soil.

**Figure 9:**
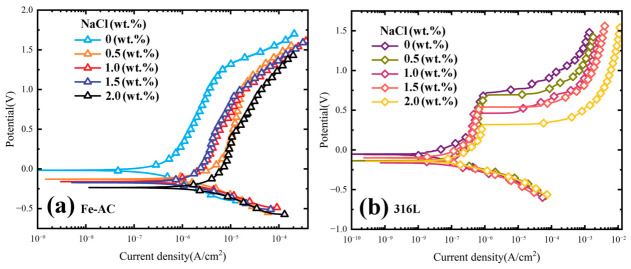
Potentiodynamic polarization curves: (**a**) Fe-AC; (**b**) 316L SS.

Figure 9 shows the potentiodynamic polarization curves of Fe-AC and 316L SS in soils with different NaCl contents, and Table 5 summarizes the corrosion potential (E_corr_), corrosion current density (Icorr), breakdown potential (Ep), and passive current density (Ip). For Fe-AC (Figure 9a), under all NaCl concentrations, the polarization curves exhibit a wide passive region, with breakdown potentials Ep as high as 1.12–1.33 V (vs. SCE), showing no obvious decrease with increasing NaCl content. The corrosion current density I_corr_ remains in the range of 0.053–0.099 μA/cm^2^, indicating an extremely low corrosion rate. This demonstrates that the high contents of Cr, Mo, W, and other elements in the amorphous coating synergistically promote the formation of a stable passive film, which effectively inhibits pitting initiation even in a high-chloride environment.

For 316L SS (Figure 9b), the polarization curves exhibit typical active–passive–breakdown behavior. In the soil with 0 wt.% NaCl, Ep is 0.539 V. When the NaCl content increases to 0.5 wt.%, Ep drops to 0.219 V; further increasing NaCl to 2.0 wt.% results in a similar Ep of 0.219 V (as listed in Table 5). More notably, the corrosion current density Icorr increases sharply from 0.604 μA/cm^2^ (0% NaCl) to 3.856 μA/cm^2^ (2.0 wt.% NaCl), an increase of approximately 6.4-fold. Meanwhile, the passive current density Ip also rises from 3.62 μA/cm^2^ to 13.25 μA/cm^2^, indicating severe degradation of the passive film protectiveness. The low breakdown potential (<0.7 V) and high corrosion current density of 316L SS reflect that its native oxide film is highly susceptible to local breakdown under chloride attack, leading to pitting corrosion.

**Figure 10:**
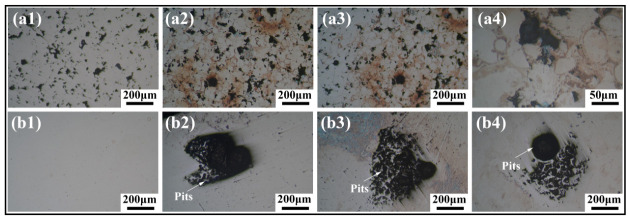
Surface morphologies of working electrodes after potentiodynamic polarization: (**a1**–**a4**) Fe-AC; (**b1**–**b4**) 316L SS.

Figure 10 shows the surface morphologies of the working electrodes after potentiodynamic polarization testing. As shown in Figure 10(a1–a4), the Fe-AC electrode surface exhibits only a dark brown oxidation discoloration, with no obvious corrosion pits or substrate exposure, indicating that the passive film formed on the coating surface was not broken down during the scan and only underwent uniform mild oxidation. In contrast, the 316L SS electrode surface in Figure 10(b1–b4) displays severe localized corrosion features: a main corrosion pit surrounded by densely distributed small pits, with the pits appearing black, indicating deep pitting. This morphology is highly consistent with the low breakdown potential, high corrosion current density, and sharp decrease in charge transfer resistance observed in EIS for 316L SS. The electrochemical test results demonstrate that the iron-based amorphous coating exhibits far superior corrosion resistance to 316L SS in chloride-containing soil. Its high breakdown potential, low corrosion current density, and stable impedance behavior are attributed to the dense passive film formed by the high Cr, Mo, and W contents in the coating. In contrast, the passive film of 316L SS is easily damaged by chloride ions, leading to high pitting susceptibility and a significantly increased corrosion rate.

### 3.4. Corrosion Mechanism

**Figure 11:**
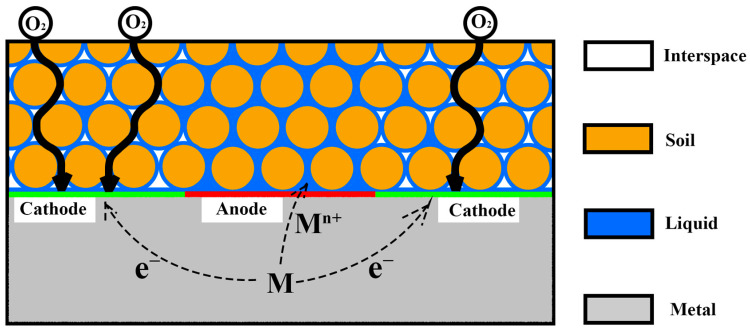
Schematic Diagram of Soil Corrosion.

As illustrated in Figure 11, soil is a complex porous medium consisting of solid, liquid, and gas phases. The liquid phase is an electrolyte solution containing soluble salts, the gas phase is mainly oxygen and carbon dioxide, and the solid phase includes mineral particles and organic matter [4,8]. Corrosion of metals in soil is essentially an electrochemical process that requires three basic conditions: an anodic reaction (metal dissolution), a cathodic reaction (reduction in a depolarizer), and an ionic conduction path. For iron-based materials, the anodic reaction is [8]:

Fe→Fe^2+^ + 2e^−^ (2)

In aerated soil, the main cathodic reaction is oxygen reduction:

O_2_ + 2H_2_O + 4e^+^→4OH^−^ (3)

Dissolved Cl^−^ in soil has a strong adsorption ability and high penetrability, which can destroy the passive film on metal surfaces and induce localized corrosion [28]. The soil used in this study, collected from the Daqing Oilfield, is salinized (pH ≈ 9.3, total salt content of 0.104 wt.%, Cl^−^ content of 0.130 wt.%). The addition of NaCl further increases the chloride concentration, thereby significantly promoting corrosion. Based on the above environmental characteristics, together with the corrosion rate, morphological observation, product analysis, and electrochemical test results, the corrosion mechanisms of 316L SS and Fe-AC in chloride-containing soil can be established, as shown in Figure 11. The marked difference in corrosion behavior between the two materials under chloride attack essentially arises from the difference in the structure and stability of their surface passive films [29].

For 316L SS, in chloride-free soil, a thin passive film composed mainly of chromium oxides can spontaneously form on its surface, leading to uniform corrosion. When chloride ions are present in the soil, Cl^−^ preferentially adsorbs at defects in the passive film (such as inclusions, grain boundaries, or scratches), displacing oxygen atoms through competitive adsorption and forming soluble complexes with metal cations, thereby inducing localized dissolution. The process can be represented as [29]:

Fe^2+^ + 2Cl^−^→FeCl_2_ (soluble) Cr^3+^ + 3Cl^−^→CrCl_3_ (soluble) (4)

Hydrolysis of these chlorides leads to local acidification:

FeCl_2_ + 2H_2_O→Fe(OH)_2_ + 2H^+^ + 2Cl^−^ CrCl_3_ + 3H_2_O→Cr(OH)_3_ + 3H^+^ + 3Cl^−^ (5)

The pH inside the pit drops significantly (to as low as 2–4), and Cl^−^ continuously migrates into the pit to maintain charge neutrality, creating an autocatalytic accelerating cycle. Once the passive film is broken through, the exposed metal substrate inside the pit acts as an anode and dissolves rapidly, whereas the large passive area outside the pit serves as a cathode for oxygen reduction, forming a “large cathode–small anode” geometry. This configuration leads to an extremely high anodic current density, driving rapid pit growth in depth [30]. XRD analysis detected that the corrosion products on 316L SS are mainly α-Fe_2_O_3_ (hematite) and γ-FeOOH (lepidocrocite), with no Cr-containing protective phases. γ-FeOOH has a loose structure and provides very poor resistance to Cl^−^ penetration. The high concentration of Fe^2+^ inside the pit diffuses outward and undergoes oxidation and hydrolysis in the soil environment around the pit opening, where the pH is relatively higher [31]:

4Fe^2+^ + O_2_ + 4H_2_O→4γ-FeOOH + 8H^+^ (6)

Some of the γ-FeOOH further dehydrates and transforms into α-Fe_2_O_3_. These products tend to accumulate around the pit opening, forming ring-like deposits, while the interior of the pit remains actively dissolving due to the acidified environment and Cl^−^ enrichment, with the pit bottom appearing black and continuously deepening. EIS showed that the charge transfer resistance Rct of 316L SS dropped sharply from 10.80 × 10^3^ Ω·cm^2^ at 0 wt.% NaCl to 2.30 × 10^3^ Ω·cm^2^ at 2.0 wt.% NaCl. The breakdown potential from polarization curves decreased from 0.539 V to 0.219 V, and the corrosion current density increased by a factor of 6.4. The macroscopic morphology revealed corrosion pits with a width of up to 3.2 mm and a depth of 3.6 mm, further confirming severe localized pitting, and the corrosion rate increased exponentially with burial time and Cl^−^ concentration (reaching 0.0562 mm/a after 55 days).

For the iron-based amorphous coating (Fe-AC), its chemical composition contains high contents of Cr (18.3 wt.%), Mo (13.7 wt.%), and W (6.0 wt.%). In the early stage of corrosion, these elements preferentially undergo anodic oxidation [20]. Because the amount of corrosion products on the Fe-AC surface is very small (insufficient for XRD detection), based on the Cr enrichment observed by EDS and the known corrosion behavior of amorphous alloys, it is inferred that an extremely thin and dense passive film consisting mainly of chromium, molybdenum, and tungsten oxides or hydroxides forms on the surface. Possible reactions include [20]:

2Cr + 3H_2_O→Cr_2_O_3_ + 6H^+^ + 6e^−^ Mo + 3H_2_O→MoO_3_ + 6H^+^ + 6e^−^ W + 3H_2_O→WO_3_ + 6H^+^ + 6e^−^ (7)

The formation mechanism of this passive film is further substantiated through a comparative analysis with other steels commonly employed in soil environments. Previous studies have reported that X52 pipeline steel exhibits a uniform corrosion rate of 0.076 mm·y^−1^ and a localized corrosion rate of 0.991 mm·y^−1^ in a soil solution containing sulfate-reducing bacteria (SRB) [32]; for Q235 carbon steel in chloride-contaminated soil, the corrosion rate increases to 0.056 mm·y^−1^ with increasing Cl^−^ concentration, accompanied by a sharp decrease in charge transfer resistance [33]; and X70 steel shows a corrosion current density exceeding 28 μA·cm^−2^ in chloride-rich soil [14]. In marked contrast, the Fe-AC developed in this study exhibits corrosion rates below 0.01 mm·y^−1^ across all Cl^−^ concentrations tested, with no pitting observed. Furthermore, its charge transfer resistance (1.16 × 10^6^ Ω·cm^2^) and breakdown potential (>1.12 V) are substantially higher than those of the aforementioned conventional steels. This pronounced disparity strongly corroborates the existence of a dense and stable Cr-rich passive layer on the Fe-AC surface, which effectively impedes the penetration and corrosive attack of Cl^−^ ions [4,34] as high as 1.16 × 10^6^ Ω·cm^2^; even when the NaCl content increased to 2.0 wt.%, Rct2 remained at 6.5 × 10^3^ Ω·cm^2^, which is higher than the Rct of 316L SS (2.30 × 10^3^ Ω·cm^2^) under the same condition. Polarization curves showed that the breakdown potential of Fe-AC is always higher than 1.12 V, and the passive current density is below 0.78 μA/cm^2^, indicating that its passive film has a wide stable potential range. Because the passive film is extremely dense, further formation of corrosion products is suppressed; therefore, only sporadic rust spots are observed on the surface, with no obvious pitting. The overall corrosion rate consistently remains below 0.01 mm/a and does not increase significantly with Cl^−^ concentration.

## 4. Conclusions

In this study, the corrosion behavior of 316L stainless steel (316L SS) and an iron-based amorphous coating (Fe-AC) buried for 15–55 days in salinized soil from the Daqing Oilfield (water content of 25%, with NaCl additions of 0, 1.0, and 2.0 wt.%) was systematically compared. The main conclusions are as follows:

The Fe-AC coating exhibits an extremely low corrosion rate (<0.01 mm/a) that remains nearly unchanged across all chloride concentrations. In contrast, the corrosion rate of 316L SS increases markedly with both chloride concentration and burial time, reaching 0.0562 mm/a after 55 days—approximately six times that of the Fe-AC—demonstrating a clear concentration-dependent and time-accumulative effect.

The Fe-AC surface shows only sporadic rust spots and no visible corrosion pits. The 316L SS surface, however, develops isolated corrosion pits with a width of 3.2 mm and a depth of 3.6 mm, surrounded by loose, iron-rich corrosion products (γ-FeOOH and α-Fe_2_O_3_) and lacking any chromium-containing protective phase.

The Fe-AC exhibits a charge transfer resistance consistently higher than that of 316L SS and a much higher breakdown potential, although its impedance decreases with increasing chloride concentration. After polarization, the Fe-AC shows only mild uniform oxidation, while 316L SS displays dense pitting with a low charge transfer resistance and a low breakdown potential.

For: 316L SS, chloride ions induce passive film breakdown and autocatalytic pitting, with loose γ-FeOOH unable to suppress corrosion. For the Fe-AC, the Cr–Mo–W synergy forms a dense oxide film that inhibits pitting initiation even though its protective impedance degrades in high-chloride soil, resulting in mild uniform corrosion rather than localized attack.

## Figures and Tables

**Table 1:** Physicochemical properties of the soil measured at a water content of 25% (wt.%).

Parameter	Total Salt Content	Cl^−^	SO_4_^2−^	CO_3_^2−^	HCO_3_^−^	pH
Soil	0.104	0.130	0.274	0.235	1.021	9.3

**Table 2:** Average corrosion rates (mm/a) of specimens buried in soils with different NaCl additions (0, 1.0, 2.0 wt.%) for 15 to 55 days.

Day	Fe-AC			316L		
	0% NaCl	1% NaCl	2% NaCl	0% NaCl	1% NaCl	2% NaCl
15	0.0068	0.0072	0.0091	0.0112	0.0124	0.0117
25	0.0071	0.0068	0.0088	0.0124	0.0134	0.0121
35	0.0082	0.0085	0.0092	0.0134	0.0210	0.0292
45	0.0084	0.0079	0.0082	0.0157	0.0254	0.0454
55	0.0074	0.0074	0.0094	0.0246	0.0296	0.0562

**Table 3:** Fitted EIS equivalent circuit parameters for Fe-AC.

NaCl Solution (wt.%)	Rs (Ω·cm^2^)	CPE1		Rct1 (×10^4^ Ω·cm^2^)	CPE2		Rct2 (×10^4^ Ω·cm^2^)
		Y01 (Ω^−1^·cm^−2^·s^−n^)	n1		Y02 (Ω^−1^·cm^−2^·s^−n^)	n2	
0	1144	5.46 × 10^−^^6^	0.78	63.06	4.33 × 10^−^^6^	0.62	116.09
0.5	869	1.24 × 10^−^^6^	0.79	56.50	5.34 × 10^−^^5^	0.64	65.24
1	606.8	1.79 × 10^−^^5^	0.73	43.95	1.29 × 10^−^^4^	0.76	24.24
1.5	641.5	1.74 × 10^−^^5^	0.73	41.49	1.05 × 10^−^^4^	0.78	35.51
2	494.4	2.74 × 10^−^^5^	0.64	34.09	6.70 × 10^−^^4^	0.75	0.65

**Table 4:** Fitted EIS equivalent circuit parameters for 316L SS.

NaCl Solution (wt.%)	Rs (Ω·cm^2^)	CPE1		R1 (×10^3^ Ω·cm^2^)
		Y0 (Ω^−1^·cm^−2^·s^−n^)	*n*	
0	1115.2	1.85 × 10^−^^5^	0.82	10.80
0.5	938.6	1.21 × 10^−^^5^	0.81	4.77
1	836.6	2.23 × 10^−^^4^	0.82	3.24
1.5	798.9	5.34 × 10^−^^4^	0.84	2.57
2	797.4	9.46 × 10^−^^4^	0.80	2.30

**Table 5:** Fitted potentiodynamic polarization parameters for Fe-AC and 316L SS.

NaCl Solution (wt.%)	Fe-AC				316L			
	E_corr_ (V)	I_corr_ (μA/cm)	E_p_ (V)	I_p_ (μA/cm)	E_corr_ (V)	I_corr_ (μA/cm)	E_p_ (V)	I_p_ (μA/cm)
0	−0.099	0.053	1.247	0.521	−0.084	0.604	0.539	3.623
0.5	−0.143	0.099	1.121	0.778	−0.086	0.709	0.219	3.580
1	−0.162	0.070	1.236	0.416	−0.059	1.066	0.452	4.474
1.5	−0.136	0.083	1.331	0.616	−0.113	1.244	0.692	5.358
2	−0.143	0.099	1.264	0.778	−0.115	3.856	0.219	13.245

## Data Availability

The original contributions presented in the study are included in the article. Further inquiries can be directed to the corresponding author.
